# H3.3 K36M Mutation as a Clinical Diagnosis Method of Suspected Chondroblastoma Cases

**DOI:** 10.1111/os.12878

**Published:** 2021-02-23

**Authors:** Haoran Mu, Yafei Jiang, Linghang Xue, Yingqi Hua, Jun Lin, Zhengdong Cai

**Affiliations:** ^1^ Department of Orthopaedics Shanghai General Hospital, Shanghai Jiao Tong University School of Medicine Shanghai China; ^2^ Departments of Pathology Shanghai General Hospital, Shanghai Jiao Tong University School of Medicine Shanghai China; ^3^ Shanghai Bone Tumor Institution Shanghai China

**Keywords:** Chondroblastoma, Chondromyxoid fibroma, Giant‐cell tumor of bone, Histone H3K36 mutation, Immunohistochemistry

## Abstract

**Objective:**

Whether H3.3 K36M mutation (H3K36M) could be an approach if the diagnosis of chondroblastoma (CB) patients was indistinct and it was suspected to be unclear clinically.

**Methods:**

We reviewed and compared our clinical experiences of CB cases and some suspected cases, which were not diagnosed distinctly, between 2013 to 2019. A total of 15 male and four female cases included in this study were seperated into two groups, CB group and suspected case (SC) group. The CB group included 13 men and 3 women, with an age range from 9 to 54 (mean age, 22 years old). The SC group included two men and one woman, with the age range from 13 to 25 (mean age, 19 years old). In both groups the patients had been followed‐up until December 2019 and none of the patients had prior treatment history. We evaluated the clinical complaints, radiological features, and clinical‐histological features of the cases and performed an immunohistochemical (IHC) study to detect whether the H3K36M expression of cases was different, consistent with a gene‐mutation analysis.

**Results:**

In both groups, the radiologic features of both groups appeared as round low‐density shadow with a clear edge, pathologic features showed diffuse proliferation of neoplastic cells with multinuclear giant cells. The radiological tumor size of CB group and SC group showed little difference, which was about 29.0*21.6 mm. Clinical‐immunohistochemical features of both groups showed chondroid matrix inside with naïve tumor cells, multinucleated giant cells, and ground substance cells. Most of them showed chondro‐related antibody positive (12 cases) but some of them showed S‐100 negative (four cases). The clear difference of both groups was the result of H3K36M IHC study and gene analysis. In our cases, the CB group showed diffuse H3K36M positive and the SC group showed negative. The gene mutation analysis revealed that H3K36M‐positive CB patients had K36M mutation, which were not found in the SC group. Sanger sequencing showed an A > T substitution at codon 36 of histone H3F3B. No other types of histone H3 mutation was detected in the CB group. Particularly, one of the suspected cases showed a G34W mutation was confirmed to be a giant cell tumor of bone (GCTB).

**Conclusions:**

Our study showed H3K36M immunohistochemistry and gene mutation analysis were specific clinical diagnostic tools to distinguish suspected CB from other giant cell‐rich or cartilage matrix‐diffuse bone tumors. The clinical‐radiological and histomorphological features of patients gave suggestions on whether the H3K36M IHC and gene analysis should be required.

## Graphical abstract

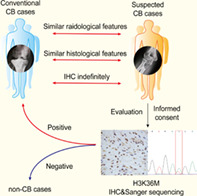

Our study showed H3K36M immunohistochemistry and gene mutation analysis were specific clinical diagnostic tools to distinguish suspected CB from other giant cell‐rich or cartilage matrix‐diffuse bone tumors. In addition, we demonstrated that the clinical‐radiological and histomorphological features of patients gave suggestions on whether the H3K36M IHC and gene analysis should be required. We suggest that further studies are needed to concern whether the application of H3K36M IHC and gene analysis will improve the patient prognosis.

## Introduction

Chondroblastoma (CB) is a rare benign chondroid tumor that composes about 1% of all bone tumors often involving the epiphysis of the long bone. It affects patients from 20 to 30 years old frequently, and it usually arises in the epiphysis or apophysis of skeletally immature patients, with a slight male predominance^1^, ^2^. CB does not often cause death of patients, but the diagnosis of CB is sometimes difficult if the suspected CB cases have an overlap of characteristics with other bone tumors, such as chondromyxoid fibroma (CMF)^3^ and giant cell tumor of bone (GCTB)^4^. The incorrect diagnosis of CB to a suspected patient will be affected more seriously if the supposed diagnosis is a malignant bone tumor.

Though most CB cases could be diagnosed through its obvious clinical‐radiological features, in some cases with similar clinical‐radiological features, methods are required to distinguish CB cases from others. To exploit more accurate diagnosis methods of CB, the difference between the generation of CB and other bone tumors has been concentrated. The previous studies have discovered it is unique that the generation of CB is associated with the mutation of histone methylation process, particularly histone H3 lysine 36 methylation (H3K36me)^5^, ^6^. H3K36me is a histone modification involved in epigenetic regulation and plays an important role in biological processes such as DNA replication, transcription, recombination, and repair of DNA damage^7^. The histone H3K36 mutation (H3K36M) dominantly inhibits H3K36me on wild‐type histones and the mutated histone H3K36 polypeptide or nucleosome can significantly down‐regulate the activity of SETD H3K36 methyltransferase, reprogramming H3K36 methylation landscape and contribute to CB generation through altering the expression of relevant tumor‐associated genes^8^, ^9^. Further histopathological studies has proved the H3K36M antibody is a sensitive and specific marker for CB^10^.

The aim of our study was to investigate whether H3K36M, a unique mutation in the process of CB generation, could recognize or exclude CB in the suspected cases and we proved this by gene analysis. Moreover, our study set out to provide an overall consideration of the suspected cases through clinical‐radiological and histopathological features to assist the clinical diagnosis. In addition, we traced the prognosis of the patients of suspected cases to evaluate the effect caused by an incorrect diagnosis of CB.

## Materials and Methods

### *Study Cohort*

The patients admitted to our hospital from 2013 to 2019 and diagnosed with CB (16 cases) or suspected CB (three cases) were included in our study and were divided into two groups, CB case group (CB group) and suspected case group (SC group) (Table 1). The CB group included 13 men and three women, with the age range from 9 to 54 (mean age, 22 years old). The SC group included two men and one woman, with the age range from 13 to 25 (mean age, 19 years old). The clinical complaints and radiological features were collected. Both groups of patients had a complaint of continuous localized pain with or without limitation. In the CB group, femur was the most frequent metastatic lesion, followed by tibia. In both groups the prognosis of patients had been followed‐up until December 2019, and none of the patients had prior treatment history. Both groups of patients were treated with tumorectomy and all of them survived. In both groups, the tumor tissues of the patients were fixed in neutral buffered formalin and processed routinely with paraffin embedding. Three pathologists examined hematoxylin and eosin‐stained glass slides from each tumor, and one paraffin block from each tumor was selected for immunohistochemistry (IHC). The known clinical‐histological features and IHC results of patients have also been collected. The present research was approved by the Institutional Research Ethics Committee of Shanghai General Hospital and informed consent was obtained from all patients.

**TABLE 1:** Clinical Summary of CB and Suspected Cases

	Histopathology	
	CB cases (n=16)	Suspected cases (n=3)
Age (year)		
Median (range)	22 (9 to 54)	19 (13 to 25)
Sex		
Male	13	2
Female	3	1
Tumor location		
Humerus	1	0
Tibia	3	0
Femur	7	1
Ulna	0	1
Rib	1	0
Talus	2	0
Phalanx	1	0
Metatarsus	1	1
Biopsy diagnosis		
Accomplished	4	1
Operation		
Tumorectomy	16	3
Prognosis		
Survival	16	3

CB, chondroblastoma.

### *Reagents and Antibodies*

For general clinical H&E staining, the H&E staining kit (Hematoxylin and eosin) (ab245880) was purchased from Abcam. For general clinical immunohistochemistry, the S100 Antibody (MA5‐12969), vimentin antibody (PA5‐27231), cytokeratin pan type I/II antibody cocktail (MA1‐82041), CD68 monoclonal antibody (MA5‐16674), alpha‐smooth muscle actin monoclonal antibody (MA1‐06110), and SOX9 polyclonal antibody (PA5‐81966) were purchased from Invitrogen, and anti‐muscle actin antibody (ab216039) and anti‐CD34 antibody (ab8158) were purchased from Abcam. For H3K36M‐antibody immunohistochemistry, anti‐histone H3 K36M antibody (ABE1447) was purchased from Millipore.

### *H&E Staining*

The tissue sections of 4 μm thickness were cut and placed on slides and were immersed in 4% paraformaldehyde for 4 h, then transferred to 70% ethanol. The sections were placed in processing cassettes, dehydrated through a serial alcohol gradient, and embedded in paraffin wax blocks. Before staining, the sections were dewaxed in xylene, rehydrated through decreasing concentrations of ethanol, and washed in PBS, and then stained with hematoxylin and eosin (H&E). After staining, sections were dehydrated through increasing concentrations of ethanol and xylene.

### *Immunohistochemistry*

Tissue sections of 4 μm thickness were cut and placed on slides. Unstained slides were incubated overnight at 37°C and subsequently deparaffinized with xylol. After washing the slides with 100% methanol, the endogenous peroxidase activity was blocked by incubating the slides for 20 min in 0.3% methanol/H2O2. Then the slides were rehydrated in 70% ethanol, 50% ethanol, and demi water. Antigen retrieval was performed by incubating the slides in boiling EDTA and followed by 2 h of cooling down in the same solution. Subsequently, the slides were blocked with PBS/1%BSA/5%milk for 30 min and incubated overnight with the first antibody in PBS/1%BSA/5% milk at 4°C. Then we used the anti‐target antibody. Slides were washed with PBS for 30 min. This was followed by a washing step with PBS for 10 min. After staining the nuclei with hematoxylin, the slides were dehydrated in demi water, 50% ethanol, 70% ethanol, and 0.3% methanol/H2O, followed by washing with xylol and mounting.

### *DNA* *Isolation and Polymerase Chain Reaction*

For DNA isolation, paraffin‐embedded tumor tissue cores were taken from the same tumor area and genomic DNA was isolated with the Chelex extraction method. The hotspot mutation containing H3F3A (128bp) and H3F3B (147bp) was amplified by polymerase chain reaction (PCR). All PCR products were obtained on a Bio‐Rad amplifier using the following program: 5 min at 95°C, followed by 40 cycles of 10 s at 95°C, 10 s at 60°C, and 10 s at 72°C, ending with a melt curve analysis.

### *Gene Mutation Analysis*

The PCR products were purified using MinElute 96 UF PCR Purification Plates (Cat. No. 28051, Qiagen) and used specific primers for Sanger sequencing. For analysis, we used the ChromasPro 2.1.8 software and Gene Runner software. Mutations were called when an aberrant peak was distinguishable from background noise in both the forward and reverse sequence or if an aberrant peak was identified by the software in at least one of the sequences and confirmed by visual inspection. Mutations in histone H3 were referred to in previous papers^6^, ^11^.

## Results

### *Clinical Radiological and Histological Features of Both Groups Showed Similarity*

**Fig. 1:**
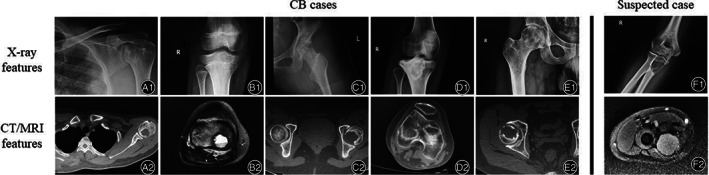
Clinical‐radiological features of CB cases and suspected cases. (**A1‐E1**) The X‐ray features of CB cases. (**F1**) The X‐ray feature of suspected case. (**A2‐E2**) The CT or MRI features of the corresponding CB cases. (**F2**) The MRI feature of the corresponding suspected case.

**Fig. 2:**
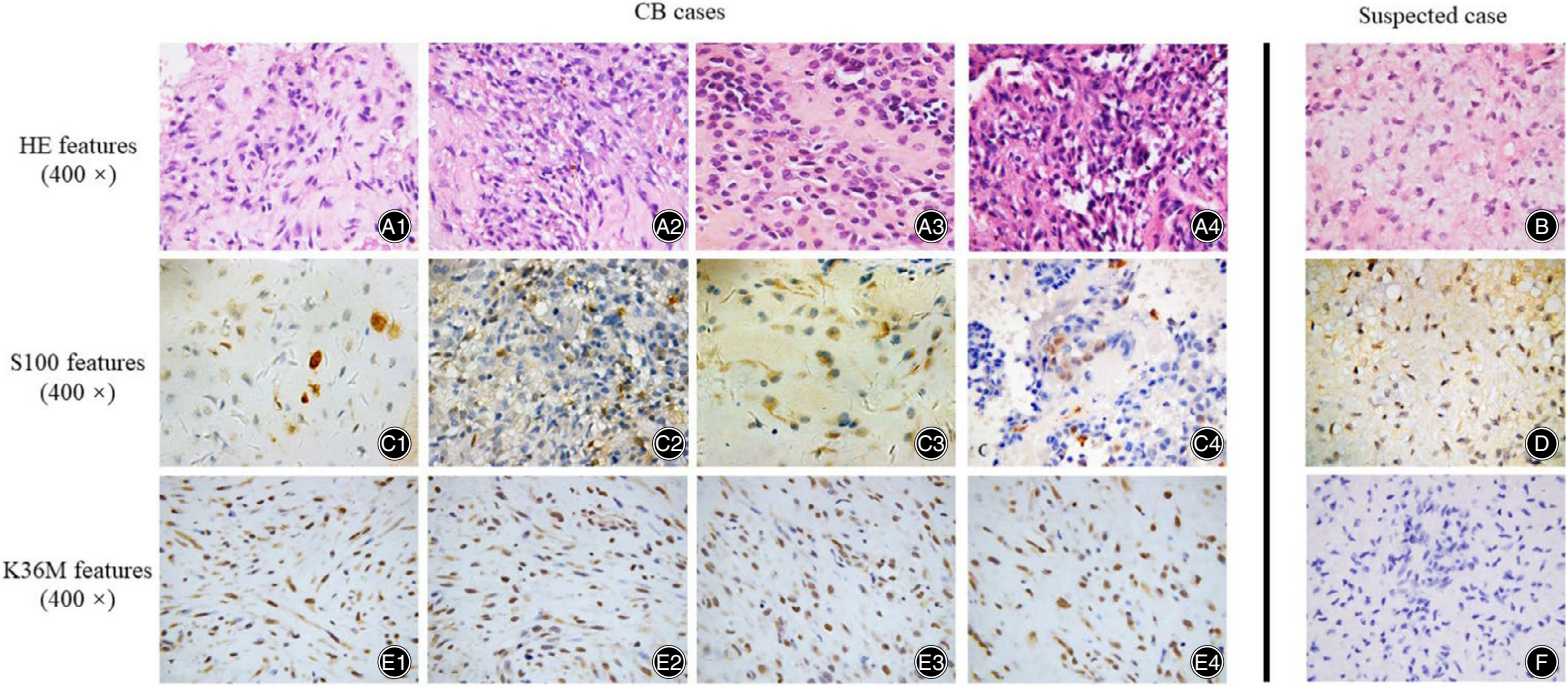
Clinical‐histological features and H3K36M IHC results of CB cases and suspected case. (**A1‐A4**) The HE features of CB cases. (**B**) The HE features of the suspected case. (**C1‐C4**) The S100 features of the corresponding CB cases. (**D**) The S100 features of the suspected case. (**E1‐E4**) The K36M features of the corresponding CB cases. (**F**) The K36M features of the corresponding the suspected case.

The radiological features of CB group and SC groups were collected, including site, the imaging tumor size, X‐ray features, computed tomography (CT) features, and magnetic resonance imagining (MRI) features (Table 2, Fig. 1). The radiological tumor size of CB group and SC group showed little difference, which was about 29.0*21.6 mm. X‐ray showed that most of the cases presented low‐density shadow (seven cases) and the edge of both groups was usually clear (five cases). The cystic bone destruction was presented in the majority of both groups in CT features (eight cases), consistent with MRI features. The histological images under microscope showed chondroid matrix inside with naïve tumor cells, multinucleated giant cells and ground substance cells (Fig. 2A1–A4 and B).

**TABLE 2:** Radiologic Summary of CB and Suspected Cases

Case	Site	Imaging size	X‐ray feartures	CT features	MRI features	
					T1WI	T2WI
CB1	Tibia	20^*^19mm	Low density shadow	Low density shadow with clear edge	Equisignal	Liquid‐liquid plane
CB2	Femur					
CB3	Femur	28^*^14mm	High density shadow with fog edge	Bright roundness with clear edge, slight bone cortex		
CB4	Femur	33^*^31mm	Low density cyst with clear edge			
CB5	Tibial	13^*^19mm	Patchy low density shadow	Bone destruction with high density spots inside		
CB6	Tibial	7^*^6mm	High density shadow			
CB7	Femur	25^*^17^*^13mm		Low density roundness with bright edge, slight bone cortex	Low signal	Highsignal
CB8	Femur	28^*^45mm		Tumor with calcified spots inside, bone destruction		
CB9	Femur					
CB10	Femur	36^*^42^*^25mm	Asymmetrical density cyst with clear edge	Cystic bone destruction with clear edge		
CB11	Talus	12^*^12.5mm	Low density shadow with high density shadow inside	Low density shadow with clear edge		
CB12	Metatarsus	37^*^20mm	Bone destruction with clear edge	Bone destruction, slight bone cortex		
CB13	Phalanx					
CB14	Talus	24^*^18^*^24mm	Low density shadow with clear edge		Highsignal	Highsignal
CB15	Humerus	29^*^24^*^30mm		Bone destruction with low densitty shadow inside	Highsignal	Highsignal
CB16	Rib			Cystic bone destruction with sclerous edge	Low signal	Highsignal
SC1	Metatarsus	15^*^10^*^24mm	Low density shadow with clear edge	Bone destruction with clear edge, slight bone cortex		
SC2	Ulna	42^*^23mm	Low density shadow with high density shadow inside		Equisignal	Equisignal
SC3	Femur	58^*^35mm	Low density shadow	Bone destruction, slight bone cortex		

CB, chondroblastoma; SC, suspected case.

### *Immunohistochemistry Showed* *H3K36M* *Positive in* *CB* *Group*

The included CB and suspected cases had already accomplished a general clinical series of IHC when the data was collected (Figs 2C1‐C4, D, and 3). The general clinical series of IHC included cartilage biomarker (S‐100), epithelial biomarker (CK), etc. Most of the patients showed as S‐100 positive (12 cases), but some of them showed S‐100 negative (four cases). In the S‐100 negative cases, one case showed SMA positive. The CB group showed diffuse H3K36M positive and the suspected cases showed H3K36M negative (Fig. 2E1‐E4, F).

**Fig. 3:**
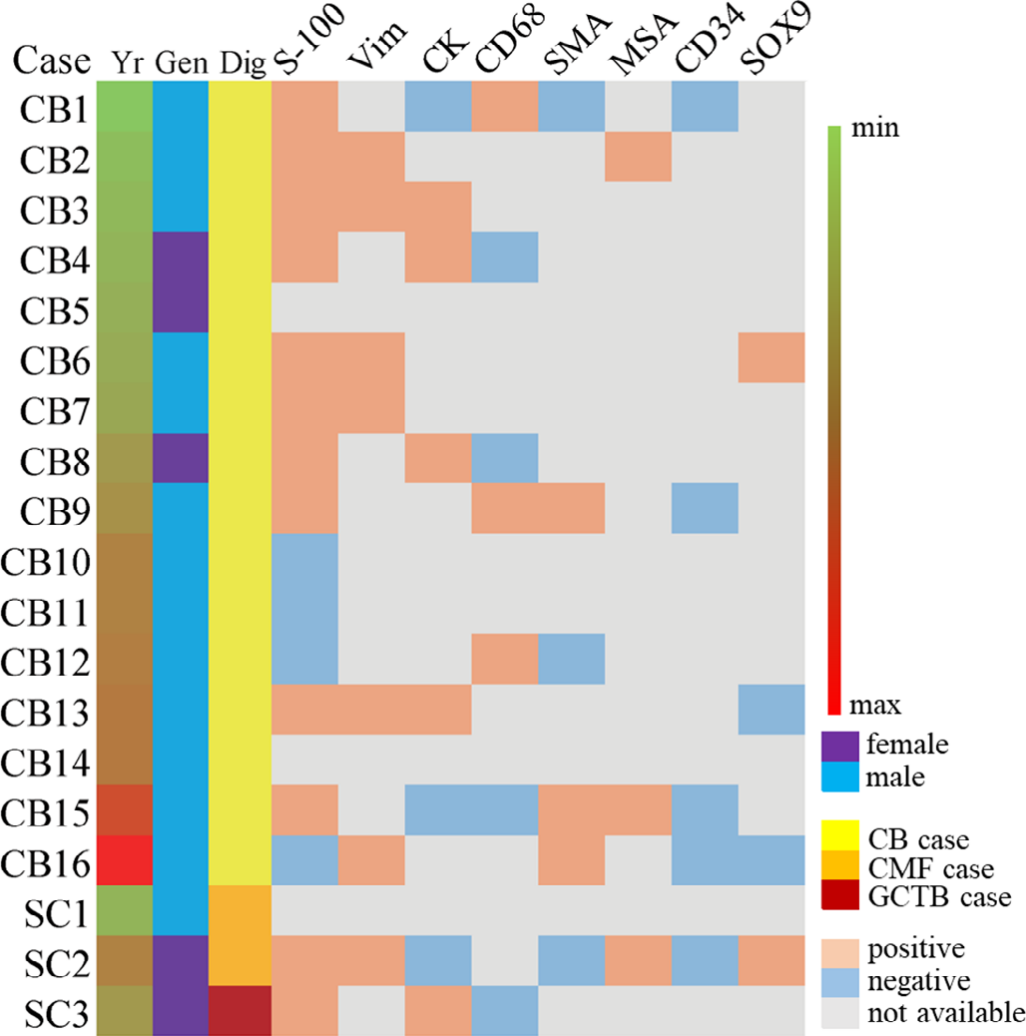
Clinical‐immunohistochemical features of CB cases and suspected cases. The diagnoses of suspected cases were confirmed by the follow‐up gene analysis. SC: suspected case.

### *Gene Mutation Analysis Re‐Confirmed the Results of* *H3K36M‐Antibody Immunohistochemistry*

**Fig. 4:**
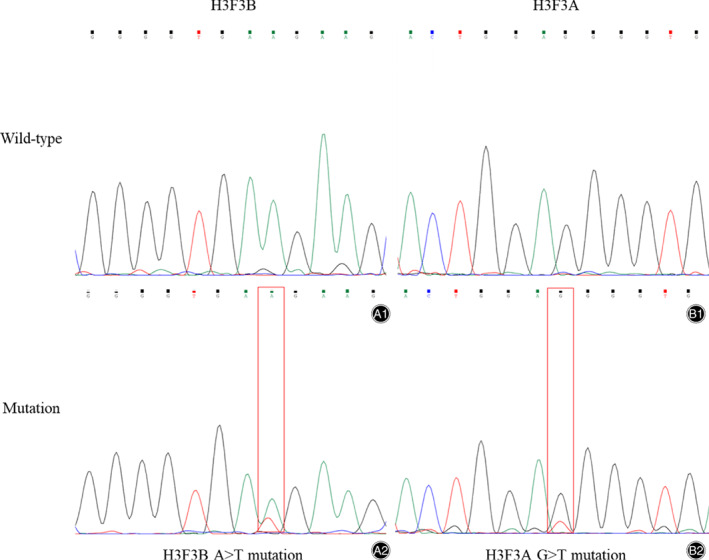
Gene mutation analysis of CB cases and suspected cases. (**A2**) Sanger sequencing showed a A > T substitution at codon 36 on histone H3F3B in CB cases. (**B2**) Sanger sequencing showed a G > T substrtution at codon 34 on histone H3F3A in SC3 (Suspected Case 3).

The gene mutation analysis revealed that H3K36M‐positive CB patients had K36M mutations, which were not found in the SC group. Sanger sequencing showed an A > T substitution at codon 36 of histone H3F3B (Fig. 4A2). No other types of histone H3 mutation were detected in the CB group. Particularly one of the suspected cases, SC3, was detected with a G34W mutation. Sanger sequencing showed a G > T substitution at codon 34 of histone H3F3A (Fig. 4B2). Therefore, the diagnosis of SC3 has been confirmed as a GCTB with a clinical following‐up for the further treatment. For the reason of no mutation having been detected in histone H3F3A and H3F3B, the other two suspected cases, SC1 and SC2, were supported to be the initial diagnosis as CMF, excluding the suspicion of CB.

## Discussion

Epigenetics has been paid attention for its critical role in the generation of some tumors, even bone tumors. Chondroblastoma (CB), as a benign chondroid tumor, sometimes is difficult to give a confirmed diagnosis because of an overlap of features with other bone tumors. Behjati *et al*.^6^ has revealed the mutations on histone H3F3A and H3F3B are the driver mutations in the generation of CB and GCTB, respectively. Previous studies have reported that the H3K36M and H3G34W mutations distinguish CB cases and GCTB cases, both in immunohistochemistry (IHC)^12^ and gene mutation analysis^13^. In our studies, we collected three suspected CB cases once treated as CB on admission, not obvious on diagnosis, and compared their results of immunohistochemistry and gene mutation analysis with the confirmed CB cases (Figs 2 and 4 E1‐E4 and F). From the compared results, a H3K36M IHC is supposed to be a more accurate method to diagnose and exclude a suspected CB case clinically and if an obvious observation of diffuse multinuclear giant cells are discovered under microscope (Fig. 2A1‐A4), a subsequent gene analysis will be suggested. Particularly, the differential diagnosis of SC3 (Suspected Case 3) of our results is an example and the detected H3G34W mutation confirmed it as a GCTB, in concordance with previous studies.

The clinic‐radiological and histological features sometimes confuse the clinical judgment^14^, ^15^. The radiological features of our cases remind this (Table 2) and only depending on the clinical radiology, the suspected cases will hidden in the CB cases with similar features as low density shadow and bone destruction. Therefore, the biopsy plays an important role in preoperative diagnosis. Nohr *et al*.^16^ have suggested the diagnostic value of H3K36M and Schaefer *et al*.^12^ have attempted H3K36M IHC in biopsy and give the conclusion that H3K36M IHC is a highly specific method in limited biopsies. Our study agrees with the previous studies that H3K36M is specific not only in giant cell‐rich bone tumors but also in cartilage matrix‐diffuse bone tumors (Figs 2 and 3). However, not all the patients will accept the preoperative biopsy (Table 1) and whether further gene analysis is needed or not after operations should be considered. In our opinion, if the radiological tumor size is large with inhomogeneous shadow and a fog edge and the histological features show giant cells rich with S‐100 positive, a further K36M IHC will be required, such as SC3 in our cohort. However, if the radiological tumor size is small with homogeneous shadow and a clear edge, such as SC1, a follow‐up will be suggested, not the further K36M IHC (Table 2, Figs 2 and 3). Substantial H3K36M data will be collected if all the patients are detected, indeed, but it will also lead to overtreatment in the social.

Previous studies have not mentioned whether H3K36M as a diagnostic method will promote patient prognosis. In our studies, whether all the patients in SC group survived though the initial diagnosis was not confirmed. From our cohort, we infer the application of H3K36M IHC and gene mutation analysis will exclude some GCTB and other bone tumors from the suspected CB cases (Figs 2, 3, 4) and improve the patient survival, though only three SC cases are lacking. Besides, H3K36M, as a driver mutation in the generation of CB, is not only a diagnostic tool but also might be explored in the therapeutic field, and next we will concentrate on its therapeutic function to improve diagnosis‐treatment integration.

In conclusion, our results show that H3K36M immunohistochemistry and histone H3F3A and H3F3B gene mutation analysis on DNA extracted from decalcified formalin‐fixed paraffin‐embedded tissue are specific clinical diagnostic tools to distinguish suspected CB from other giant cell‐rich or cartilage matrix‐diffuse bone tumors. Although H3K36M IHC and gene mutation analysis are specific, not all the patients are suggested to receive and an overall consideration with clinical‐radiological features and histomorphological features of the cases should be considered. Which groups of patients need to be paid close attention is still a question. Future studies like the multi‐center or single‐center trial to reveal whether the application of H3K36M IHC and gene analysis will improve the patient prognosis are necessary to prove our results.

## Declarations

### *Ethics Approval and Consent to Participate*

All procedures performed in studies involving human participants were in accordance with the ethical standards of the institutional and/or national research committee and with the 1964 Helsinki Declaration and its later amendments or comparable ethical standards.

### *Consent for Publication*

Informed consent was obtained from all individual participants included in the study.

### *Authors' Contributions*

H Mu analyzed and interpreted the patient data and was a major contributor in writing the manuscript. Y Jiang, J Lin, Y Hua, and Z Cai collected the patient data and L Xue and Z Chen helped to complete this work. All authors read and approved the final manuscript.
